# Granulomatosis with polyangiitis in a patient with polydipsia, facial nerve paralysis, and severe otologic complaints: a case report and review of the literature

**DOI:** 10.1186/s13256-022-03492-7

**Published:** 2022-07-28

**Authors:** Lukas Koenen, Ulf Elbelt, Heidi Olze, Sören Zappe, Steffen Dommerich

**Affiliations:** 1 Department of Otolaryngology, Head and Neck Surgery, Charité-Universitätsmedizin Berlin, Mittelallee 2, 13353 Berlin, Germany; 2 Department of Medicine B-Hepatology, Gastroenterology, Endocrinology, Diabetes, Hematology, Oncology, Palliative Care, Brandenburg Medical School, Neuruppin, Germany

**Keywords:** Granulomatosis with polyangiitis (D014890), Hearing loss, sensorineural (D006319), Hypopituitarism (D007018), Diabetes insipidus (D003919), Facial paralysis (D005158)

## Abstract

**Background:**

Granulomatosis with polyangiitis, formerly known as Wegener granulomatosis, is a necrotizing vasculitis with granulomatous inflammation that belongs to the class of antineutrophil cytoplasmic antibodies-positive diseases. It occurs in a localized and a systemic form and may present with a variety of symptoms. Involvement of the upper respiratory tract is very common, while neurologic, endocrinological, and nephrological dysfunction may occur.

**Case presentation:**

We describe the case of a 29-year-old Central European male patient presenting with severe bilateral sensorineural hearing loss, otorrhea, and one-sided facial nerve paralysis. The patient was unsuccessfully treated with i.v. antibiotics at another hospital in Berlin, and tympanic tubes were inserted. After presentation to our emergency room, he was hospitalized and further diagnostics started. Increased fluid intake and 12 kg weight gain over the last months were reported. The patient was diagnosed with granulomatosis with polyangiitis and diabetes insipidus. The patient’s condition improved after treatment with rituximab.

**Discussion:**

A comprehensive PubMed search of all articles with granulomatosis with polyangiitis and diabetes insipidus was conducted to assess which combination of symptoms occurs simultaneously and whether other parts of the pituitary are commonly involved. The 39 selected articles, describing 61 patients, showed that ear–nose–throat involvement occurred most commonly, in 71% of cases. Of patients, 59% had involvement of the anterior pituitary gland, while true panhypopituitarism occurred in 13% of cases. Only one case report featured the same set of symptoms as described herein.

**Conclusion:**

Granulomatosis with polyangiitis is a highly variable disease, commonly involving the upper airways, but that may present with symptoms solely related to the pituitary gland. Clinicians should have a low threshold to investigate for granulomatosis with polyangiitis in patients with therapy-resistant otorrhea. Patients may present with a complex set of symptoms, and integrating different specialists when additional symptoms occur may lead to faster diagnosis.

**Supplementary Information:**

The online version contains supplementary material available at https://doi.org/10.1186/s13256-022-03492-7.

## Background

Granulomatosis with polyangiitis (GPA) is a disorder formerly known as Wegener granulomatosis. Its hallmark is necrotizing vasculitis with granulomatous inflammation. It involves primarily small and medium-sized vessels with a predilection for the upper and lower airways. Necrotizing glomerulonephritis is common [1]. The current definition was formed in 2012 by the Chapel Hill Conference for Consensus Criteria [2].

GPA belongs to a group of anti-neutrophil cytoplasmic antibody (ANCA)-positive diseases called ANCA-associated vasculitis, although ANCA testing may be negative in 10–20% of cases [3].

Research led to the identification of different subsets of ANCA-positive diseases. Anti-proteinase 3 activity (PR3-ANCA) is most common in GPA, while patients with microscopic polyangiitis and Churg–Strauss syndrome are most often anti-myeloperoxidase ANCA (MPO-ANCA) positive [4].

GPA occurs in a systemic and a localized form. ENT involvement occurs in up to 90% of individuals. The sinonasal area is most commonly affected [5–7]. Otologic involvement is the second most common presentation [8, 9]. Localized forms of GPA with involvement of the ear and/or the upper respiratory tract have been described, especially in younger patients [10]. Dysfunction of the central nervous system is common at some point of the disease; the result can be dysfunction of cranial nerves II, VI, and VII, but also endocrine involvement of the pituitary gland [11–14]. Severe headache is the most common nonendocrine symptom of hypophysitis. Chiasmal syndrome and ocular paresis may occur due to the increased sellar mass. Isolated DI centralis may occur as an endocrine symptom and is characterized by deficient secretion of arginine vasopressin (AVP), leading to clinical symptoms of polyuria and polydipsia. More frequently, combined hormonal insufficiency of the anterior and posterior pituitary occurs. Hypogonadotropic hypogonadism, secondary hypothyroidism, and potentially life-threatening secondary adrenal insufficiency are the most frequent endocrine disorders of the anterior pituitary, at least in patients with primary hypophysitis, in contrast to the finding of vulnerable gonadal and growth hormone axis and more robust adrenal and thyroid axes in patients with pituitary adenoma [15]. However, data on hypophyseal function of patients with secondary hypophysitis due to GPA are scarce.

We present the case of a patient with localized, severe granulomatosis with polyangiitis with otitis media, facial nerve palsy, and pituitary dysfunction resulting in DI.

This case is presented according to the CARE guidelines [16].

Additionally, a comprehensive systematic review of existing literature was performed to examine how often and to what extent hypophyseal dysfunction and ENT involvement occur in patients with GPA.

## Case presentation

**Fig. 1:**
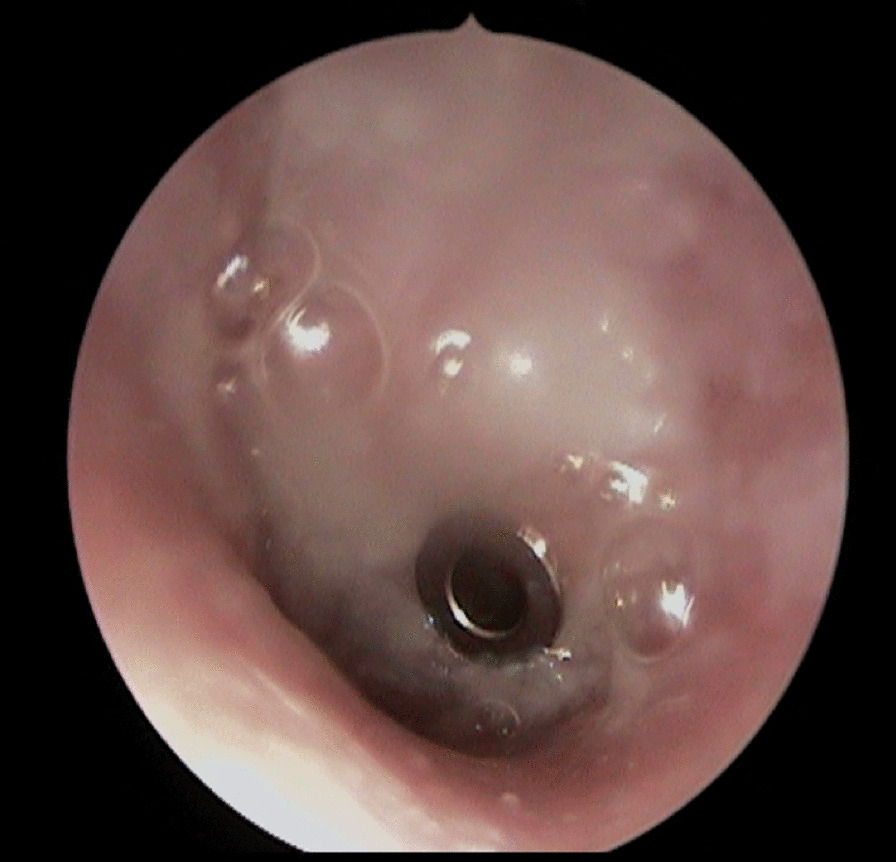
Left ear of the patient 1 day after presentation to our clinic with visible serous otorrhea after insertion of Titan tubes

**Fig. 2:**
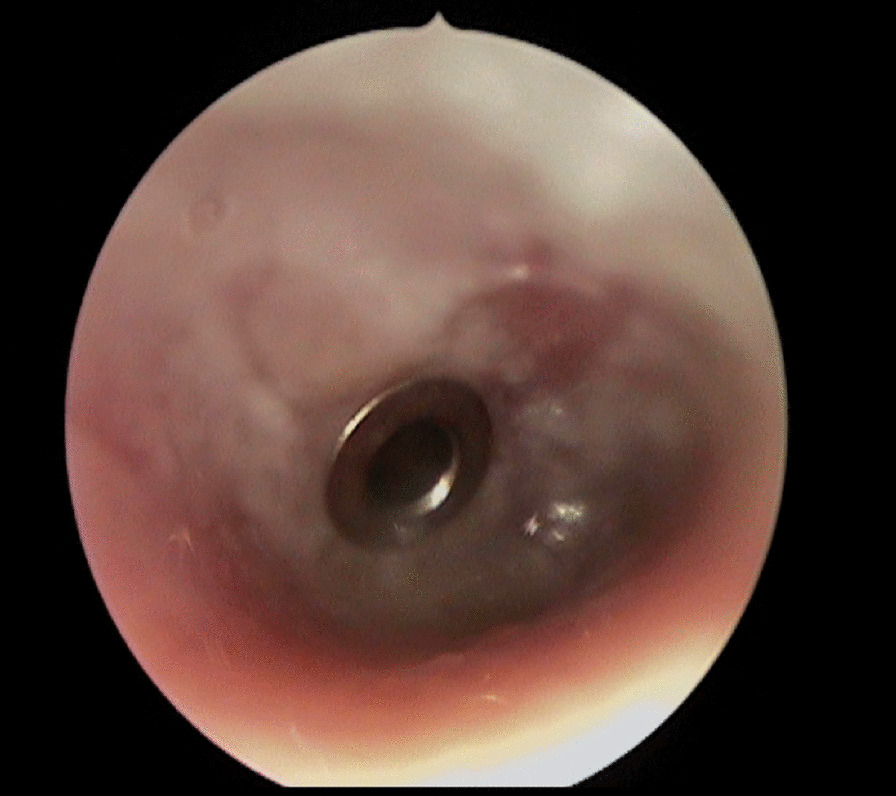
Right ear of the patient 1 day after presentation to our clinic with visible serous otorrhea after insertion of Titan tubes

**Fig. 3:**
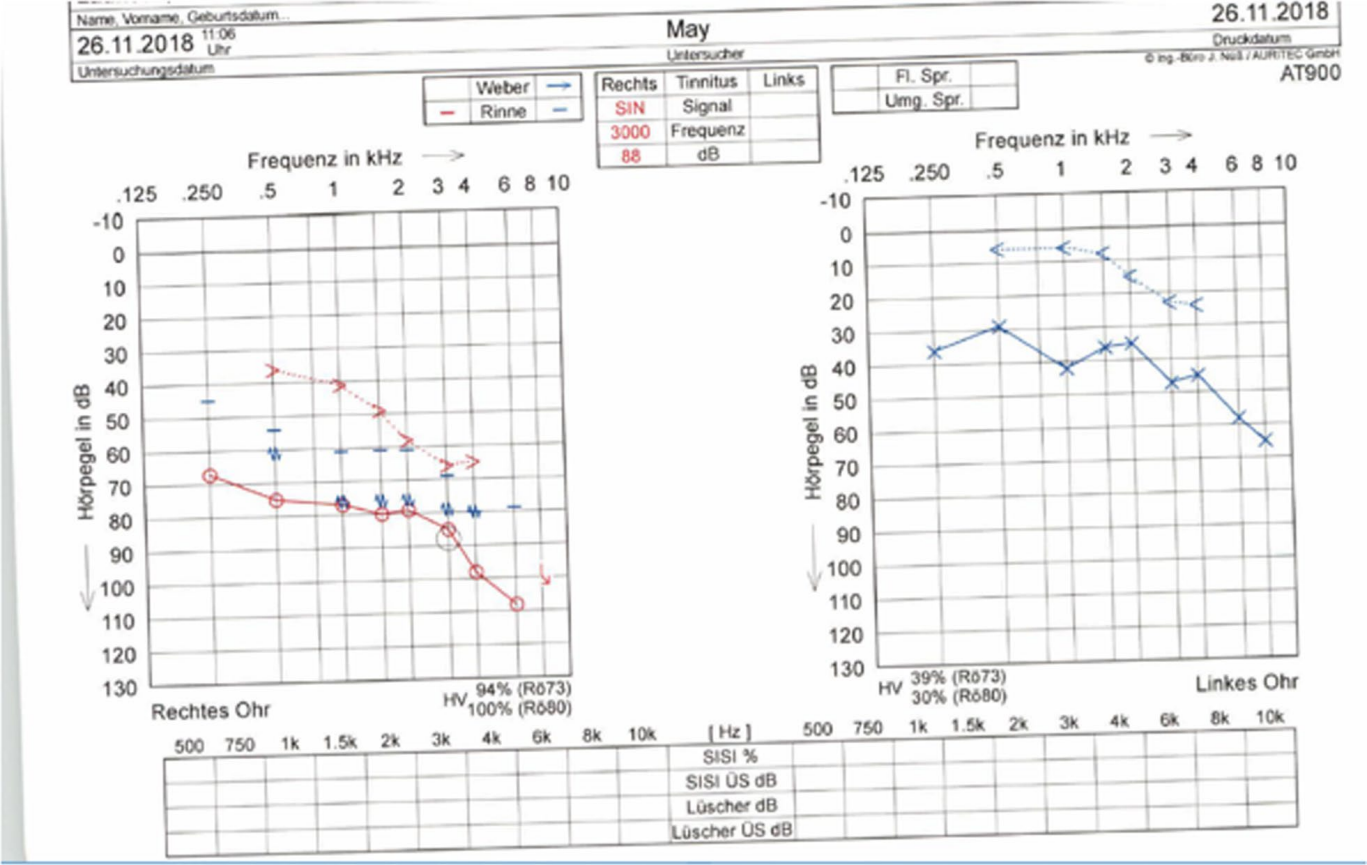
Audiometric analysis on 26/11/2018, showing mixed hearing loss on the right side with Fletcher index of 80 dB and conductive hearing loss on the left side with Fletcher index of 40 dB

The patient is a white German male of 29 years of age who presented with otalgia and serous otorrhea (Figs. 1, 2) to the otolaryngology resident on call in the ER of the Charité University Hospital in Berlin in November 2018. The patient reported having had these symptoms for 1 month. He had visited an otolaryngology clinic in October 2018 with facial nerve palsy with House–Brackmann score (HB) < III. At the clinic, treatment was started with i.v. antibiotics and prednisolone as well as tympanocentesis with insertion of T-tubes. Audiometric analysis revealed combined sensorineural and conductive hearing loss on the right side and conductive hearing loss on the left side (Fig. 3). A computed tomography (CT) scan report on his temporal bone described fluid retention on the mastoid on both sides, possibly with osteolysis. Routine laboratory analysis including human immunodeficiency virus and hepatitis showed slightly elevated C-reactive protein (34.9 mg/l) but no further anomalies. On microbiologic testing, the middle ear fluid revealed biological flora without detection of pathogens.

Facial nerve function normalized and otorrhea decreased after receiving treatment. The patient was discharged after 7 days with middle ear tubes in situ.

Upon presentation to the ER, facial nerve function was still abnormal with HB score of II, and extensive serous otorrhea persisted. The middle ear ventilation tubes were in situ. The patient also reported increased fluid intake of 9 L per day and weight gain of 12 kg in 7 weeks.

Laboratory analysis showed positive c-ANCA at 87.0 U/ml with increased anti-PR3 activity in combination testing. Anti-MPO activity was negative.

On the basis of this testing, a diagnosis of granulomatosis with polyangiitis was established.

**Fig. 4:**
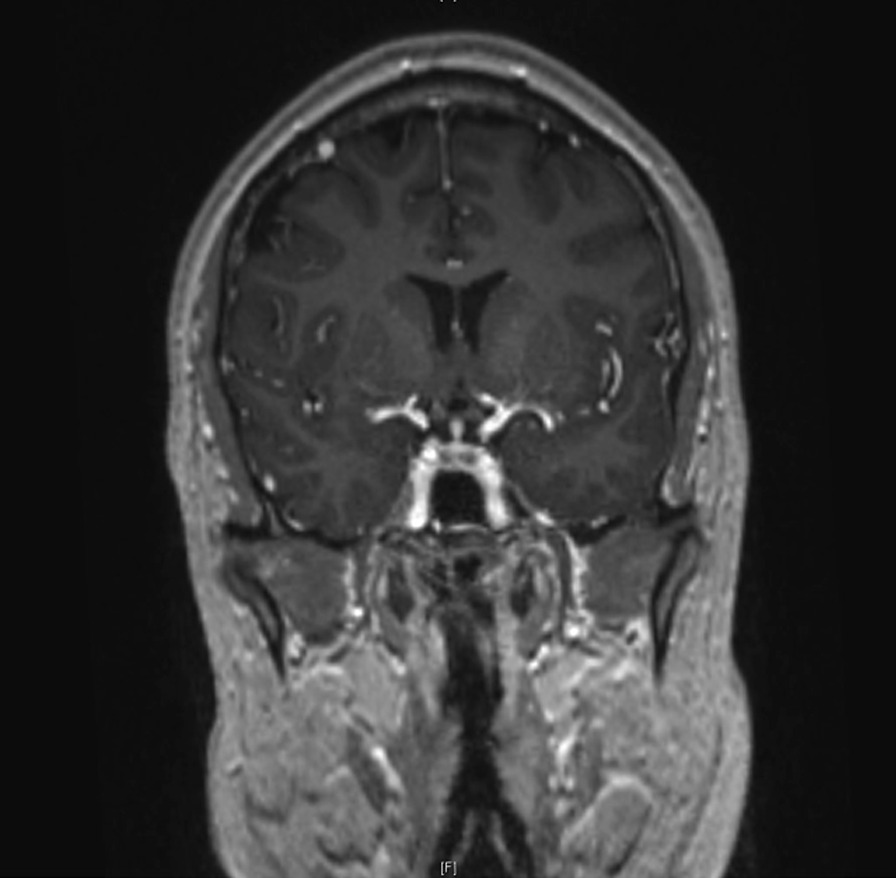
Coronal imaging of the hypophysis on a 3-T T1-weighted MRI image

**Fig. 5:**
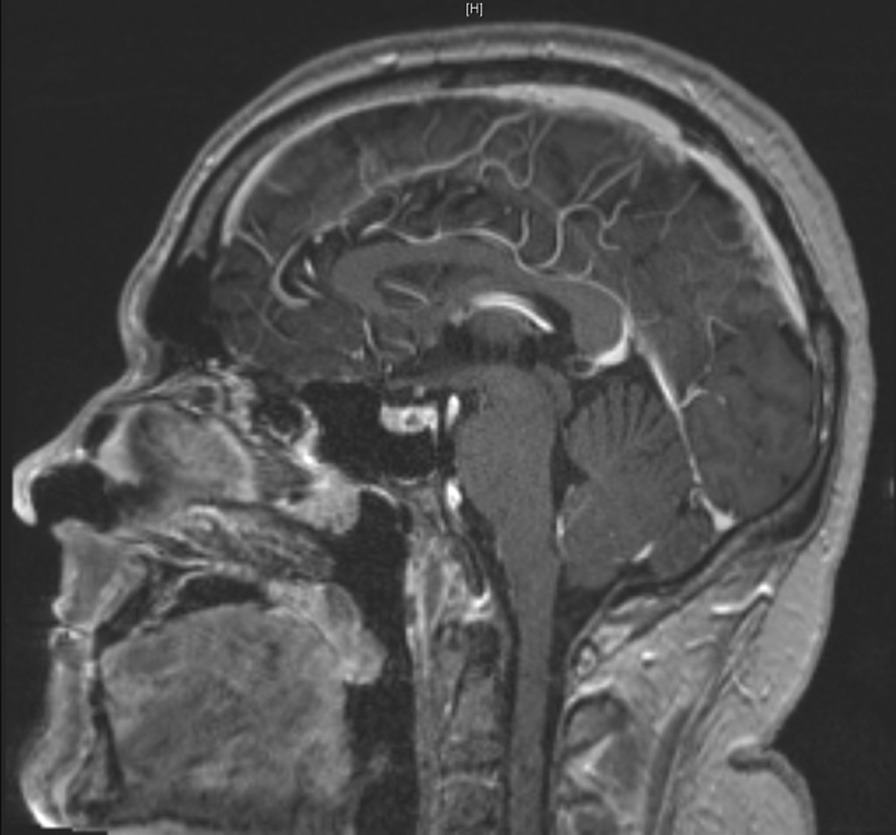
Saggital imaging of the hypophysis on a 3-T T1-weighted MRI image

A magnetic resonance imaging (MRI) scan of the brain showed slight widening of the pituitaries infundibulum and unclear inhomogeneity between the anterior and posterior pituitary (Figs. 4, 5).

Clinical and laboratory evaluation of the anterior pituitary function did not indicate insufficiency of the somatotropic, gonadotropic, thyrotropic, or corticotropic axis. In addition, serum prolactin was within the reference range.

Plasma sodium concentration was 144 mmol/l with plasma osmolality of 300 mosm/kg. In a water deprivation test, urine osmolality increased insufficiently from 131 to 418 mosm/kg. Measurement of hypertonic saline-stimulated plasma copeptin (2.63 pmol/l) confirmed the diagnosis of DI.

Treatment for GPA was successfully started with 1 g rituximab i.v., and ANCA values decreased to 22.2 U/ml at 4 months after discharge and finally to 13.4 U/ml in October 2019 (Additional file 1).

## Discussion

GPA is a variable disease that may present with a complex set of symptoms [11–14]. The patient in our case presented with otalgia, otorhea, facial nerve dysfunction, hearing loss, and polydipsia. Upon presentation to our department, the posterior pituitary gland function was already affected. While the resulting increase in fluid intake was not seen as a burden by the patient, medical history showed that symptoms of DI had existed before otologic involvement. Referral to specialists in endocrinology and rheumatology was sought after the diagnosis of GPA.

A comprehensive systematic PubMed search was performed using the search term ‘Wegener granulomatosis AND pituitary dysfunction OR granulomatosis with polyangiitis AND pituitary dysfunction OR Wegener granulomatosis AND diabetes insipidus OR granulomatosis with polyangiitis AND diabetes insipidus’. The article references were scanned for additional literature, resulting in a total of 65 articles. All case reports with confirmed diagnosis of GPA in addition to diabetes insipidus were included. Articles published in foreign language (other than English or German) and for which no full text was available were excluded. In total, 39 articles were selected for analysis after applying inclusion and exclusion criteria.

The 39 selected articles described 61 different cases for analysis (Table 1). ENT involvement in patients with GPA was very common, with 43 patients reported to have any ENT area affected (72%). Sinonasal disease was very common, with 21 patients affected (35%). Otologic complaints were less common, being reported in nine patients (15%). Two of the selected case series, representing a total of 17 patients, did not specify the involved subarea. This may result in underestimation of the total percentage of patients affected per subarea.

**Table 1:** Published articles with patients matching our inclusion criteria, with publication date and site of ENT involvement

Author	Year of publication	ENT involvement
Garovic *et al.* [17]	2001	No
Roberts *et al.* [18]	1995	Case 1: no Case 2: sinusitis
Katzman *et al.* [19]	1999	Case 1: sinonasal congestion Case 2: stridor, laryngeal stenosis, septal perforation, saddle-nose deformity
Hajj-Ali *et al.* [20]	1999	Sinusitis, epistaxis
Muir *et al.* [21]	2004	Ear infection (not defined) with cyst
Düzgün *et al.* [22]	2005	Dry oral mucosa
Hurst *et al.* [23]	1983	Sinusitis
Haynes *et al.* [24]	1978	No
Czarnecki *et al.* [25]	1995	Sinusitis, septal perforation with saddle-nose deformity
Roberts *et al.* [18]	1995	No
Bertken *et al.* [26]	1997	Sinusitis, epistaxis, external otitis
Katzman *et al.* [19]	1999	Sinusitis
Miesen *et al.* [27]	1999	No
Goyal *et al.* [28]	2000	No
Seror *et al.* [29]	2006	Case 1: sinusitis Case 2: gingivitis
Špíšek *et al.* [30]	2005	Epistaxis
McIntyre *et al.* [31]	2007	No
Yong *et al.* [32]	2008	Sinusitis and epistaxis
Cunnington *et al.* [33]	2009	Case 1: epistaxis, nasal crusting Case 2: otalgia, otorrhea, hearing loss (not specified)
Xue *et al.* [34]	2009	No
Barlas *et al.* [35]	2011	Sinusitis
Santoro *et al.* [36]	2011	No
Tenorio Jimenez *et al.* [37]	2011	Sinusitis, septal perforation, saddle-nose deformity
Hughes *et al.* [38]	2012	No
Pereira *et al.* [39]	2013	No
Kapoor *et al.* [40]	2014	Eight cases with ENT involvement (unspecified)
Peters *et al.* [41]	2018	Epistaxis, nasal congestion, recurrent bilateral otitis media, hearing loss
Ohashi *et al.* [42]	2017	Sensorineural hearing loss, otitis media, facial nerve paralysis
Esposito *et al.* [13]	2017	Case 1: no Case 2: serous otitis media Case 3: no
Xie *et al.* [43]	2017	No
Byrne *et al.* [14]	2016	Sinusitis
Eli *et al.* [44]	2016	No
Vandergheynst *et al.* [45]	2015	Sinusitis
De Parisot *et al.* [11]	2015	Six of nine cases with ENT involvement (unspecified)
Bando *et al.* [46]	2015	Saddle-nose deformity
Sampei *et al.* [47]	2014	Sinusitis
Slabu *et al.* [48]	2013	Sinusitis
Van Durme *et al.* [49]	2011	Sinusitis, otitis media
Dutta *et al.* [50]		Case 1: otorrhea, hearing loss Case 2: nasal blockage, bilateral ear blockage

Of the 61 cases, 36 (59%) experienced at least one symptom of anterior pituitary dysfunction. Hyperprolactemia was the most common hormonal dysbalance (*n* = 20, 33%), while hypothyroidism (*n* = 15, 25%) and hypogonadism (*n* = 14, 23%) ranked second and third. Panhypopituitarism was relatively uncommon and occurred in eight (13%) patients.

Most of the patients examined in this study had an established diagnosis of GPA before developing symptoms suggestive of DI (*n* = 33, 54%). The majority of patients (*n* = 35, 57%) developed ENT symptoms suggestive of GPA earlier than symptoms suggestive of DI. In 11 cases of this subgroup (31%), the ENT symptoms occurred days to months before DI symptoms. In 18 cases (51%), ENT symptoms developed years prior to diagnosis. Six cases (17%) did not include a timeframe for the occurrence of the symptoms.

DI was diagnosed before the diagnosis of GPA in 17 cases (28%). In this subgroup, the symptoms of DI developed days to month before diagnosis of GPA in three (18%) cases and years before diagnosis of GPA in another three (18%) cases. The other cases did not offer a timeframe (Table 2).

**Table 2:** Evaluation of pituitary function tests and latency of occurrence of symptoms per case

Author	Pituitary function	First manifestation and latency
Garovic *et al.* [22]	Decreased FSH and LH (on estrogen replacement therapy), decreased prolactin, decreased TSH (on thyroid hormone replacement)	Not mentioned
Roberts *et al.* [23]	Low TSH, low FSH and low LH, low cortisol, DI	Four-month history of deteriorating vision with bitemporal hemianopia; 2 months later DI
	DI, no ant. pituitary deficiency	Cheek and temporal pain, left-sided hearing loss, sinusitis; several days later DI
Katzman *et al.* [24]	Increased prolactin	Latency unclear
	Increased prolactin, rest normal	Nasal symptoms before pituitary gland symptoms, latency unclear
Hajj-Ali *et al.* [25]	Not mentioned	Two month sinusitis; later DI
Muir *et al.* [26]	Not mentioned	Ear infection and DI at same time
Düzgün *et al.* [27]	Ant. pituitary hormones normal	Otitis media 2 months before diabetes symptoms
Hurst *et al.* [28]	Not tested	Polyarthritis, 3 months later serous otitis media, 6 months later sinusitis
Haynes *et al.* [29]	Not tested	No ENT involvement
Czarnecki *et al.* [30]	Hyperprolactemia (not biochemical proven)	Hyperprolactemia and DI 3 years after general ENT symptoms
Roberts *et al.* [23]	Not tested	Not mentioned
Bertken *et al.* [31]	Luteinizing hormone response to gonadotropin-releasing hormone and the thyroid-stimulating hormone response to thyrotropin-releasing hormone were blunted	Frontal headaches, rhinorrhea, epistaxis, anosmia, amenorrhea, and weight loss. One month later: cushingoid appearance, tenderness of the left maxillary sinus, reduced pubic hair, and reduced olfaction
Miesen *et al.* [32]	Hyperprolactemia, low serum testosterone	Polyuria and polydipsia as presenting symptoms
Goyal *et al.* [33]	Hypothyroism	Several-year history of GPA, polyuria, polydipsia, lethargy, and headaches
Seror *et al.* [34]	Pituitary hormones normal	1987 bloody-crusty rhinitis, septum necrosis, saddle-nose deformity, arthralgia; 2002 for polydipsia and polyuria, diagnosis DI established
	Corticotropic, gonadotropic, and thyrotropic deficiency	First diagnosis of GPA in 1995 with crusty rhinitis, nasal septum necrosis, mouth ulcers. In 2000, presentation with galactorrhea
Špíšek *et al.* [35]	Panhypopituitarism, low adrenocorticotropin-releasing hormone (ACTH), thyroid-stimulating hormone (TSH), follicle-stimulating hormone (FSH), and luteinizing hormone (LH) and deficit of peripheral hormones. Insulin test further revealed growth hormone deficiency, low insulin-like growth factor (IGF)-I concentration → anterior and posterior pituitary insufficiency	2002 skin abscesses, weight loss (15 kg in 3 months), collapses, and erectile dysfunction. Later epistaxis and headaches (no timeframe mentioned)
McIntyre *et al.* [36]	Ant. pituitary insufficiency (low TSH, low prolactin, low LH, low FSH, low estradiol, low cortisol	Collapse due to hypothermia, hypoglycemia, and hypotension, transsphenoidal biopsy was taken, then third nerve palsy right. Five months later, central DI. Six months later, left hemiparesis, sensorineural hearing impairment. Diagnosis established. Patient deceased 13 months after initial presentation Necrotizing scleritis of both eyes
Yong *et al.* [37]	DI; further pituitary function tests normal but secondary adrenal insufficiency and secondary hypogonadism	Polydipsia and polyuria as presenting sign with central DI established, otitis media, and thickening of sphenoid and maxillary mucosa found
Cunnington *et al.* [38]	No ant. pituitary dysfunction	July 2000, epistaxis, haemoptyis, nasal crusting, vasculitic skin rash, and bilateral episcleritis; no specific diagnosis yet. May 2005, polyuria and polydypsia; diagnosis of DI and GPA
	No pituitary dysfunction	1995, GPA with malaise, nose bleeds, and sinusitis; 2004, diagnosis of DI
	Prolactin and thyroid function normal, other values tampered by prednisolone and oral anticonception	2002, otalgia, otorrhea, and hearing loss, treatment for GPA started. In 2003, polyuria, polydipsia, and frontal headaches; diagnosis of DI
Xue *et al.* [39]	LH, FSH, GH, and TSH normal	Intermittent fever and polydipsia, insensibility of her lower extremities, pitted edema on face and lower extremities since 0.5 year. Five months later, polydipsia, diagnosis of DI and GPA
Barlas *et al.* [40]	High prolactin	Polydipsia, polyuria, sinusitis at presentation
Santoro *et al.* [41]	Low LH and FSH, slightly elevated TSH	Previous diagnosis of GPA. At presentation, arthralgia and skin ulcer; 3 months later, fever, cough, and sinusitis; 2 months later, diagnosis of DI
Tenorio Jimenez *et al.* [42]	Secondary hypothyroidism and hypogonadism	15-year history of GPA. At presentation, n. VI palsy, headache, and diplopia. Diagnosis of DI
Hughes *et al.* [43]	Panhypopituitarism	2008, initial diagnosis with uveitis and scleritis. 2011, polydipsia, polyuria, head ache, and fatigue; diagnosis of DI
Pereira *et al.* [44]	Hyperprolactemia, hypothyroidism, probable DI	Several-year history of GPA. Hyponatremia at presentation; 4 months later, bitemporal superior quadrantanopia, diagnosis of DI
Kapoor *et al.* [45]	7/8 secondary hypogonadism, 6/8 DI, 4/8 secondary hypothyroidism, 1/8 secondary arenal insufficiency, 2/8 panhypopituitarism, 1/8 hyerprolactemia, 2/8 hypoprolactemia	4/8 with DI as presenting symptom, the rest developed after diagnosis of GPA. Latency not mentioned
Peters *et al.* [46]	Hyperprolactemia, hypothyroidism, DI	ENT symptoms at presentation. Diagnosis of GPA, 1 year later with cranial nerve palsies
Ohashi *et al.* [47]	Only DI	2011, diagnosis of GPA with ENT symptoms. 2012, polydipsia and polyuria; diagnosis of DI
Esposito *et al.* [20]	Normal ant. pituitary function at diagnosis; 2 years later, secondary hypogonadism and GH deficiency	Diagnosis of GPA with ENT symptoms. Four months later, polydipsia and polyuria; diagnosis of DI
	DI, no ant. pituitary deficiency	Polydipsia and polyuria. Four months later, ENT symptoms; diagnosis of DI
	DI, no ant. pituitary deficiency	Sinusitis, otitis media. Two months later, polydipsia and polyuria; diagnosis DI
Xie *et al.* [48]	DI, no test of ant. pituitary function mentioned	Polydipsia and polyuria as presenting sign
Byrne *et al.* [21]	DI, no test of ant. pituitary function mentioned	Presentation with nasal congestion and headache. Four months later, FESS due to sinusitis. One year later, polydipsia, polyuria, and diplopia
Eli *et al.* [49]	DI, hyperprolactemia	Presentation with galactorrhea and amenorrhea, 1.5 years after fatigue and arthralgias and hemoptysis and shortness of breath; 3.5 years after initial presentation, diagnosis
Vandergheynst *et al.* [50]	DI, ant. pituitary function normal	Polydipsia and polyuria and chronic sinusitis at presentation; diagnosis of DI
De Parisot *et al.* [18]	DI in 7/9, 7/9 hypogonadism, 5/9 TSH deficiency, 4/9 hyperprolactemia, 2/9 GH deficiency, 1/9 ACTH deficiency	Pituitary disease diagnosed after GPA in 8/9 patients at median of 58.5 months, concomitant in one case
Bando *et al.* [51]	DI, GH deficiency	Chronic sinusitis, COM, auditory disturbance when 34 years of age. With 38 years of age, nasal stiffness, fatigue, appetite loss, saddle-nose deformity. At 43, polydipsia and polyuria
Sampei *et al.* [52]	DI, no test of ant. pituitary function mentioned	DI 4 months before admission, headache and right-sided loss of vision and sinusitis at admission
Slabu *et al.* [53]	DI, hyperprolactemia, hypothyroidism, low GH	Longstanding diagnosis of GPA; at diagnosis, polydipsia and polyuria
Van Durme *et al.* [54]	DI, hypogonadotropic hypogonadism, hyperprolactemia, primary hyperthyroidism	Nausea and vomiting at presentation, history of sinusitis and otitis media,
Dutta *et al.* [55]	DI, no ant. pituitary dysfunction	Polyuric syndrome at presentation, 1 year later fever, rash, and arthralgias; 3 years later, ear discharge, decreased hearing, nasal and oral ulcers
	DI, no ant. pituitary dysfunction	Nasal blockage, cough, fever, polyuria, and bilateral ear blockage since 5 months; central DI and GPA established

FSH - Follicle-stimulating hormone, LH – Luteinizing hormone, TSH - Thyroid-stimulating hormone, DI – Diabetes Insipidus, ACTH - Adrenocorticotropin-releasing hormone, IGF - Insulin-like growth factor, GH – Growth hormone

Among the reviewed articles, only one report matched our patient’s complaints of therapy-resistant otitis media, facial nerve paralysis, sensorineural hearing loss, and DI [42]. Although GPA can occur as a very limited localized disease, e.g., as antibiotic-resistant otitis media with or without mastoiditis [51–53], we want to highlight that GPA can also occur with a combination of involved localized areas, as described herein. The current case, as well as the cases reviewed, highlight the complexity of this disease.

This review supports previous research and suggests that more than 70% of patients with GPA and DI have symptoms in the field of otolaryngology [6]. We found that ENT-related symptoms might occur more often before symptoms of DI. These results might explain why early involvement of ENT specialists was found to result in substantially increased survival [54].

In line with our findings, in many of the cases reviewed, patients experienced ENT symptoms at least 1 year before symptoms of DI occurred. Sinonasal disease is the most common manifestation in the head and neck area, but other symptoms occur frequently. The otolaryngologist has to manage acute and chronic symptoms, so knowledge about the different forms of the disease is fundamental.

The specific enlargement of the pituitary infundibulum on MRI is the result of involvement of the stalk and the hypothalamus in patients with DI [55, 56]. These images allow for distinction from other pathologies affecting the gland. While panhypopituitarism occurred in only a few patients, our study suggests that anterior pituitary dysfunction is slightly more common than anticipated before [27, 29]. Even with these numbers, it may be possible that gland dysfunction is underdiagnosed due to the blunted response when treated with corticosteroids, which most patients with GPA receive during the course of their disease. Hyperprolactemia, hypothyroidism, and hypogonadism are the most common abnormalities in our study. Care by the attending physician in regard to possible dysfunction of pituitary gland function is important, especially because several patients developed DI years after diagnosis of GPA. If polydipsia and polyuria are present, tests for anterior pituitary gland function must be performed.

## Conclusion

This case demonstrates a relatively rare occurrence of DI in a patient with GPA and demonstrates the difficulty of diagnosing the disease. All healthcare professionals involved in the diagnostic process of the disease must have knowledge about its possible variable course. This is especially important since delayed diagnosis can lead to significant morbidity and possibly mortality, while appropriate treatment options exist [57].

## Supplementary Information

**Additional file 1. **Flowchart.

## Data Availability

All data generated or analyzed during this study are included in this published article and its additional information files.

## Abbreviations

GPA: Granulomatosis with polyangiitis
ANCA: Antineutrophil cytoplasmic antibodies
ENT: Ear–nose–throat
PR3-ANCA: Anti-proteinase 3 antineutrophil cytoplasmic antibodies
MPO-ANCA: Anti-myeloperoxidase antineutrophil cytoplasmic antibodies
AVP: Arginine vasopressin
ER: Emergency room
HB: House–Brackmann score
CT: Computed tomography
IGF: Insulin-like growth factor
LH: Luteinizing hormone
FSH: Follicle-stimulating hormone
T3: Triiodothyronine
T4: Thyroxine
ACTH: Adrenocorticotropin
DI: Diabetes insipidus
