# Supplementary material for: Granulomatosis with polyangiitis in a patient with polydipsia, facial nerve paralysis, and severe otologic complaints: a case report and review of the literature
Source: J Med Case Rep. 2022 Jul 28;16:291. doi: 10.1186/s13256-022-03492-7 (PMC9331564; doi:10.1186/s13256-022-03492-7)
Supplement: Supplementary file 1 — Additional file 1. Flowchart. [file 13256_2022_3492_MOESM1_ESM.docx]

Flow Diagram/Timeline

October 25^th^, 2018: Visit to Emergency Room at Vivantes Klinikum Neukölln (VKN)

October 10^th^ – November 1^st^ Hospitalisation at VKN
-> paracentesis and bilateral middle ear tubes
-> i.v. treatment with antibiotics and prednisolone
-> combined sensorineural and conductive hearing loss
-> CT imaging: massive fluid retention in mastoid possibly with osteolysis
-> laboratory tests including HIV and Hepatitis: negative
-> microbiology testing results negative for bacteria

November 13^th^, 2018: Visit to Emergency Room at VKN

November 9^th^ and 14^th^: Microbiologic testing results from ear swabs return negative

November 15^th^: First visit to emergency department at the Charité University Hospital (CUH)

November 16^th^: Visit to the outpatient department Otolaryngology and hospitalisation for further diagnostics

November 18^th^: c-ANCA results return positive

November 20^th^ : Consultation with Departments for Rheumatology and Endocrinology

January 6^th^: Discharge from CUH
